# Measuring Motivation for Cognitive Effort as State

**DOI:** 10.3389/fpsyg.2021.785094

**Published:** 2021-12-09

**Authors:** Max Blaise, Tamara Marksteiner, Ann Krispenz, Alex Bertrams

**Affiliations:** ^1^Educational Psychology Lab, Institute of Educational Science, University of Bern, Bern, Switzerland; ^2^Department of Educational Psychology, School of Social Sciences, University of Mannheim, Mannheim, Germany

**Keywords:** cognitive motivation, effort, need for cognition, psychometrical scale, measurement

## Abstract

People's motivation to engage in cognitive effort is a variable which is relevant in different psychological domains (e.g., social cognition research). Despite its potential benefits, a psychometrically sound state measure of cognitive motivation is still lacking. We therefore developed the 10-item motivation for cognition (MFC) state scale based on the established conceptualization and measure of trait need for cognition (NFC). In two studies, we examined the psychometric properties of the new measure. Study 1 revealed that the MFC scale reliably measures a one-dimensional construct. Moreover, the MFC scale was related to NFC and choice of task effort in an expected manner. In Study 2, relationships with NFC, achievement motives, self-control capacity, subjective vitality, momentary affect, and choice of task effort provide further preliminary support for the MFC scale as being a valid measure of momentary cognitive motivation. We discuss the utility of the new scale in psychological research and practice.

## Introduction

In modern times people must process complex information almost daily. Individuals are required, for example, to resolve mental tasks assigned at work or in academic contexts, to make decisions based on sellers' or politicians' claims, or to organize their and their families' prosperity. As various dual-process theories propose (see Evans, 2008), information can be encountered and processed in roughly two ways—either effortlessly (automatic, reflexive, heuristic) or effortfully (controlled, reflective, analytic). How much cognitive effort people tend to invest in processing information has crucial implications for what they achieve, the decisions they make, how they actively search for information in social settings, and even how well they are emotionally adjusted (Preckel et al., 2006; Bertrams and Dickhäuser, 2009, 2012; Fleischhauer et al., 2010; Carnevale et al., 2011; Curşeu, 2011; Harman, 2011; Meier et al., 2014). Consistently, individual differences in the motivation to expend cognitive effort have been a subject of rigorous psychological research (for a review, see Cacioppo et al., 1996). The present work deals with the measurement of such cognitive motivation. Specifically, we aim at supplementing the existing measures of respective individual differences with a state measure that is still lacking.

Research has frequently shown that the extent to which people apply cognitive effort in a given situation depends on various factors. For instance, when distracted, or when their self-regulatory resources are depleted, people are unlikely to spend cognitive resources on in-depth processing of current information; they rather tend to use ways of information processing that require low effort, such as applying heuristics and stereotypes (e.g., Newman, 1996; Dudley and Harris, 2003; Pohl et al., 2013). Motivation is another crucial determinant of how intensely individuals engage in effortful cognitive processes. In various psychological realms, including educational psychology (e.g., Preckel et al., 2006; Bertrams and Dickhäuser, 2009; Meier et al., 2014), consumer psychology (e.g., Zhang, 1996; Drolet et al., 2009), and social cognition (e.g., Dudley and Harris, 2003; Tormala and Clarkson, 2008), motivation for cognitive effort has often been examined by relying on individual differences in need for cognition (NFC).

Need for cognition refers to stable individual differences in people's tendency to engage in and enjoy effortful cognitive activity (Cacioppo and Petty, 1982). Across situations, people high in NFC are motivated to invest cognitive effort; for example, when considering arguments (Cacioppo et al., 1986), when choosing from tasks of different cognitive difficulty (Bertrams and Dickhäuser, 2010; Kramer et al., 2021), and when completing cognitively challenging tasks (Unnikrishnan Nair and Ramnarayan, 2000; Rudolph et al., 2018). Cacioppo et al. (1996) extensive review of the literature revealed that people high in NFC actually spend more cognitive effort in an array of cognitive challenges. In contrast, people low in NFC display a relative absence of engagement in and enjoyment of cognitive effort (Cacioppo et al., 1996). In terms of teaching and learning, several findings of the recent years contribute to a better understanding of NFC and its role in the classroom. For instance, students low in NFC are more likely to experience math anxiety, which in return, is related to poorer math performance (Maloney and Retanal, 2020). When it comes to reading and memorizing, learners high in NFC rely on elaborate learning strategies even in the absence of explicit instruction, whereas individuals low in NFC benefit more from these strategies than their peers high in NFC (Schindler et al., 2019). Recently, numerous other variables in student populations have been of particular interest in NFC research, e.g., in student teachers (Grass et al., 2018) or in third to ninth graders (Luong et al., 2017); underlining its relevance for educational psychology as well.

In order to tap NFC, the NFC scale (embedding a brief version) has been developed and found to have good psychometric properties (Cacioppo and Petty, 1982; Bless et al., 1994; Cacioppo et al., 1996; German adaptation: Bertrams and Dickhäuser, 2010; Preckel, 2014). The NFC scale assesses individuals' general tendency to be motivated to engage in cognitive effort across various situations; that is, NFC is conceptualized and measured as a relatively stable individual difference (Cacioppo et al., 1996). However, we are not aware of an existing state measure of NFC. Such a state measure would be a useful tool, as indicated by research on affect and emotion where the trait–state distinction in variables and their measures is well-established. Prominent examples are the Positive and Negative Affect Schedule (Watson et al., 1988), the State-Trait Anxiety Inventory (Spielberger et al., 1970), and the Subjective Vitality Scales (Ryan and Frederick, 1997). Traits may be considered as dispositions which are there all along but only manifest from time to time in reaction to relevant situations, whereas states may be seen as concrete occurrences (Fridhandler, 1986; Spielberger and Vagg, 1995). According to Fridhandler (1986), the concepts of state and trait basically differ on the dimensions of (a) short vs. long duration, (b) continuousness vs. reactivity, (c) concreteness vs. abstractness, and (d) situational vs. personal causality. Thus, the trait–state distinction is important for accurate measurement, depending on whether dispositions or actual momentary experiences in specific situations are the focus of interest. We propose that this may apply to motivation for cognitive effort, too.

Though NFC, measured as a trait, has been found to predict behavior in concrete situations (see Cacioppo et al., 1996), a respective state measure, completed in temporal proximity to the assessment of the interesting criterions, may even more reliably disclose expected relationships. Consistently, we assume that researchers and practitioners would benefit from a reliable and valid state measure of motivation for cognitive effort. For example, Fleischhauer et al. (2015) argue that their repeated measurements of participants' NFC self-concept might have induced NFC state effects. In this case, it could make sense to control for any unintended side effects by applying a NFC-related state measure.

We therefore developed a state measure of motivation for cognitive effort based on the German version (Bless et al., 1994) of Cacioppo and Petty (1982) NFC (trait) scale. The German NFC scale has been found to be a reliable and valid measure (Bless et al., 1994; Bertrams and Dickhäuser, 2010), and was hence a suitable basis for this endeavor. In addition to displaying good psychometric properties, we intended the new scale to consist of no more than 10 items. The reason for this was that we expect a state measure of cognitive motivation to be primarily applied in experiments or else in the field, where time for data collection is usually scarce. Recent research has shown the psychometric usability of even much shorter self-report measures (Gogol et al., 2014).

For developing a 10-item state scale, we first adapted the instruction and items of the German NFC scale (Bless et al., 1994) for assessing a momentary motivational state. Then, we selected 10 items based on content- and language-related grounds.

Afterwards, in Study 1, we examined the new state scale's factorial structure and psychometric properties (inner consistency, validity). The conceptually closely related NFC scale has usually been considered as one-factorial (e.g., Cacioppo and Petty, 1982; Bertrams and Dickhäuser, 2010), but diverging structures also have been received and discussed (Tanaka et al., 1988; Davis et al., 1993). For this reason, and because the state scale was a completely new measure, we preferred exploratory over confirmatory factor analysis. Moreover, for initial validation purposes, we examined the relation between the new state scale and the NFC scale. We expected both measures to be strongly positively related because they refer to the same construct (i.e., cognitive motivation). Furthermore, we hypothesized that the new state scale would be positively related to the cognitive effort one would momentarily be willing to invest in a cognitive task. Since deciding whether to engage in a challenging task highlights the volitional aspects inherent in the concept of effort, task-choice procedures indicate the willingness to exert cognitive effort (Westbrook and Braver, 2015). Findings from studies on cognitive effort discounting showed that people avoid cognitively demanding tasks based on subjective cost calculations (e.g., Westbrook et al., 2013). It seems that individuals high in NFC are less likely to avoid, respectively, are more motivated to engage in cognitively demanding activities than people low in NFC. As our new state measure can be understood as a more proximal measure of cognitive motivation, we predicted that responses obtained from the new state scale are related to task choice over and above trait NFC.

In Study 2, we aimed to further investigate the validity of the new state measure. For this purpose, we tested whether the state scale is theoretically meaningfully related to several trait and state measures. As in Study 1, we assessed NFC and expected the state scale responses to be positively related to NFC. In addition, we applied a measure of dispositional achievement motives, specifically, *hope of success* and *fear of failure*. Hope of success is an approach tendency and fear of failure an avoidance tendency with respect to achievement situations (Clark et al., 1956; Lang and Fries, 2006). As achievement often requires cognitive effort, people higher compared to lower in hope of success may more likely feel motivated to engage in cognitive effort, expressing their propensity to approach achievement situations. The contrary may apply with regard to fear of failure and the expression of avoidance of achievement situations. Thus, we assumed higher state scale responses to be associated with higher hope of success and lower fear of failure, respectively. Whether people engage in effortful cognition or rely on effortless heuristics has been shown to depend on their current *self-control capacity* (Masicampo and Baumeister, 2008; Pocheptsova et al., 2009; Pohl et al., 2013). Moreover, cognitive motivation has been found to decrease with lowered self-control capacity (Finkel et al., 2006). Based on these previous findings, we predicted the new state scale measure and state self-control capacity to be positively related. The state scale should be positively related to current *subjective vitality* as well. Subjective vitality is typically associated with high intrinsic motivation (Kasser and Ryan, 1996; Ryan and Frederick, 1997). As NFC is considered a kind of intrinsic motivation (Cacioppo et al., 1996), so should its state counterpart. Based on similar grounds—that is, the established relation between intrinsic motivation and *positive affect* (e.g., Gillet et al., 2013)—we also expected people with higher responses to the new state scale to experience higher momentary positive affect. As suggested by recent research (Gillet et al., 2013), state cognitive motivation as an intrinsic motivation may be merely weakly negatively related to momentary *negative affect*. Furthermore, as in Study 1, we examined whether higher values on the new state scale predict choice of cognitively more demanding task options. Both the state scale as well as the task choice are considered to be proximal state measures of cognitive motivation and, thus, conceptually closely related. Therefore, we assumed that the relationship between the state scale responses and task choice would hold even when the other applied trait variables (addressing the trait–state distinction) and state variables (addressing the discriminant validity) were controlled for.

## Development of the State Measure

In order to assess a state rather than a trait, we rephrased the instruction and the 34 items of the German NFC scale (Bless et al., 1994). The changes were kept to a minimum. Afterwards, two experts and one non-expert in theory and findings on NFC appraised the new items with respect to whether they are suitable for measuring state motivation for cognitive effort. All three raters evaluated 14 items concordantly as suitable. Through discussion, out of these 14 items, the 10 best fitting items were selected. Selection of items was based on keeping the breadth of the construct, and whether content and language were in line with capturing a momentary state. In the following, we will refer to the new 10-item state scale as motivation for cognition (MFC) scale. The scale name was chosen because in classical motivation research, the term “motivation” refers to current motivational states as opposed to dispositional “needs” or “motives.”

## Study 1

### Methods

#### Participants

The participants were 294 university students (73% female; *M*_age_ = 22.11 years, *SD*_age_ = 2.88) from two universities in Southern Germany. We excluded one additional participant who obviously did not follow the instruction of the MFC scale (the participant crossed out the instruction “at the moment” and changed it to “in general”).

The students were recruited on campus or in lectures and asked to complete a brief paper-pencil questionnaire. By the time both studies were conducted, it was neither compulsory nor customary at the university where the studies were conducted to seek explicit ethical approval for a study asking for participants' self-reports on MFC, NFC, and task choice. Nevertheless, we carefully ensured that Study 1 and Study 2 were conducted in line with the ethical guidelines of the American Psychological Association (APA) and in full accordance with the ethical guidelines of the German Association of Psychologists (DGPs). In particular, we did not induce any negative states in the participants. Hence, we had no reasons to assume that our study would induce any negative states in the participants exceeding the normal risks of filling out a questionnaire. Also, written informed consent was obtained according to the guidelines of the German Psychological Society. Informed consent included information about (a) research object, (b) study procedure, (c) duration and allowance, (d) possible benefits of participation, (e) anonymity of data collection, and (f) possible risks of participation. Further, all participants were explicitly informed that participation was voluntary and could be terminated at any time without any reason or negative consequences for the participant. Participants had to declare that they were at least 18 years old, had read the informed consent, and agreed to the rules of participation.

#### Measures

We did not apply any additional measures in the present study than the ones mentioned in the following. The order of the three measures within the questionnaire was shuffled across participants. On its title page, the questionnaire also contained questions on personal data (age, gender, course of study). The Cronbach's αs and descriptive statistics in the present study are presented in Table 1 and the Results section.

**Table 1:** Item wordings and descriptive statistics of the motivation for cognition (MFC) scale in study 1.

**Item**	**Wording (English/*German*)**	***M***	***SD***	**λ**	***r*_it_**
1.	Right now, I would really enjoy a task that involves coming up with new solutions to problems. *Die Aufgabe, neue Lösungen für ein Problem zu finden, würde mir im Moment wirklich Spaß machen*.	0.19	1.64	0.64	0.59
2.	Right now, I would prefer a task that is intellectual, difficult, and important to one that is somewhat important but does not require much thought. *Ich würde im Moment lieber eine Aufgabe lösen, die Intelligenz erfordert, schwierig und bedeutend ist, als eine Aufgabe, die zwar irgendwie wichtig ist, aber nicht viel Nachdenken erfordert*.	−0.17	1.57	0.58	0.54
3.	If I read something that confuses me right now, I would just put it down and forget it. (R) *Wenn ich jetzt etwas lesen würde, das mich verwirrt, dann würde ich es zur Seite legen und vergessen*. (R)	0.23	1.81	0.55	0.52
4.	Right now, the notion of thinking abstractly is not appealing to me. (R) *Abstrakt zu denken, reizt mich gerade nicht*. (R)	0.07	1.74	0.60	0.57
5.	Right now, I prefer to think about small, daily projects to long-term ones. (R) *Ich mag im Moment lieber über kleine, alltägliche Vorhaben nachdenken, als über langfristige*. (R)	−0.26	1.82	0.57	0.54
6.	Right now, I would rather do something that requires little thought than something that is sure to challenge my thinking abilities. (R) *Ich mag im Moment lieber etwas tun, das wenig Denken erfordert, als etwas, das mit Sicherheit meine Denkfähigkeit herausfordert*. (R)	−0.08	1.73	0.80	0.74
7.	Right now, I would like to avoid situations where there is a likely chance I will have to think in depth about something. (R) *Ich möchte jetzt gerade Situationen vermeiden, in denen die Wahrscheinlichkeit groß ist, dass ich intensiv über etwas nachdenken muss*. (R)	−0.04	1.69	0.84	0.78
8.	Right now, I would like to solve a puzzle. *Jetzt gerade würde ich gerne eine knifflige Aufgabe lösen*.	−0.86	1.62	0.74	0.68
9.	Right now, I prefer complex to simple problems. *In diesem Moment ziehe ich komplizierte Probleme einfachen Problemen vor*.	−0.99	1.54	0.66	0.61
10.	Right now, I would enjoy thinking about an issue even when the results of my thought would have no effect on the outcome of the issue. *Es würde mir im Moment Spaß machen, über ein Problem nachzudenken, sogar dann, wenn die Ergebnisse meines Denkens keinen Einfluss auf die Lösung des Problems hätten*.	−0.57	1.69	0.58	0.54
	MFC scale	−0.25	1.17		

*N = 294. λ, factor loading; r_it_, corrected item-total correlation; (R), item has to be recoded. Means were calculated after the respective items had been recoded. Items were responded on a scale from −3 (does not apply at all) to +3 (applies exactly). Exact wording of the instruction: “Please indicate how far the following statements apply to you personally in this moment”*.

##### Motivation for Cognition

State cognitive motivation was measured with our newly developed MFC scale. Participants answered each of the 10 items (e.g., “Right now, I prefer complex to simple problems”; for the wordings of all items, see Table 1) on a seven-point Likert-type scale from −3 (*does not apply at all*) to +3 (*applies exactly*). The instruction asked the participants to respond to the items as they applied in the present moment (i.e., “Please indicate how far the following statements apply to you personally *in this moment*”).

##### Need for Cognition

We used the established German brief version of the NFC scale (Bless et al., 1994). The brief scale consists of the 16 items from the German 33-item NFC scale that had the highest factor loadings in Bless et al. (1994). Participants completed the 16 items (e.g., “I tend to set goals that can be accomplished only by expending considerable mental effort”) on seven-point Likert-type scales from −3 (*does not apply at all*) to +3 (*applies exactly*). Participants were instructed to indicate on the items how each statement applies to them in general.

##### Choice of Task Difficulty Item

Participants were asked to indicate which difficulty level they would choose for a completely unspecified task that would immediately follow the present questionnaire. They could choose one of six different difficulty levels: Level 1 was described as requiring very low cognitive effort, level 2 as requiring low cognitive effort, level 3 as requiring somewhat low cognitive effort, level 4 as requiring somewhat high cognitive effort, level 5 as requiring high cognitive effort, and level 6 as requiring very high cognitive effort. Thus, the higher the chosen difficulty level was, the higher the cognitive effort one was motivated to exert in the present situation. Similar measures for state task motivation have been used in previous research (Finkel et al., 2006; Bertrams and Dickhäuser, 2010). We used the number of the chosen difficulty level for data analyses. Actually, the participants did not receive any task after finishing the questionnaire. A translated version of this measure is presented in Appendix A.

### Results and Discussion

#### Factor Structure

The Kaiser–Meyer–Olkin measure of sampling adequacy (Kaiser, 1970), *KMO* = 0.90, and the statistical significance of Bartlett (1954) test of sphericity, *p* < 0.001, indicated that the present data were suitable for factor analysis. First, we determined the number of factors by applying the scree test (Cattell and Vogelmann, 1977) and the minimum average partial (MAP) test (Velicer, 1976). As the MAP test is superior to the scree test in terms of objectivity and reliability (O'Connor, 2000), we intended to weigh the solution from the MAP test more strongly. To run the (revised) MAP test, we used O'Connor's SPSS syntax (retrieved from https://people.ok.ubc.ca/brioconn/nfactors/nfactors.html on September 23, 2014; see also O'Connor, 2000). Afterwards, we conducted a principal axis factor analysis, as recommended by Russell (2002).

Both the results of the scree test and the MAP test indicated a one-factor solution for the MFC scale (initial eigenvalues: 4.90, 1.22, 0.73, 0.65, 0.58, 0.54, 0.46, 0.39, 0.28, 0.26). The subsequent principal axis factor analysis revealed that the extracted single factor explained a total of 43.71% of the variance of MFC. All 10 items loaded sufficiently high (defined as λ > 0.32; Tabachnick and Fidell, 2007) on this factor, λs > 0.54 (see Table 1). Thus, it emerged that MFC, as measured by the MFC scale, is a one-dimensional construct.

#### Descriptive Statistics and Reliability

Table 1 provides means and standard deviations for the single items and the total scale. Moreover, the corrected item-total correlations are given, which were all reasonably high, *r*_it_s >0.51. Visual inspection of the scale value-frequency histogram and the normal Q–Q plot of the scale values revealed that the total scale values were normally distributed. There were no outliers in the present sample because no total scale value exceeded the critical *z*-value of ±3.29, *z*_min_ = −2.35, *z*_max_ = 2.17 (for testing for normality and outliers, see Tabachnick and Fidell, 2007). The inner consistency of the scale can be considered as high, Cronbach's α = 0.88, speaking for the reliability of the MFC scale. Furthermore, the average inter-item correlation of 0.43 was slightly outside the interval of [20, 40] recommended by Briggs and Cheek (1986), suggesting that the MFC scale measures a quite homogenous construct.

#### Validity

As expected, MFC correlated positively with NFC, *r* = 0.51, *p* < 0.001, two-tailed (descriptive statistics for NFC in the present sample: *M* = 0.88, *SD* = 0.78, α = 0.85). Although this correlation can be considered as high (Cohen, 1988), it is far from determining that MFC and NFC are identical. Moreover, in accordance with our prediction, MFC was positively correlated with chosen task difficulty, *r* = 0.56, *p* < 0.001, two-tailed (descriptive statistics for the task choice item in the present sample: *M* = 3.46, *SD* = 1.06).

Next, applying multiple regression analysis, we regressed task choice on MFC and NFC as simultaneous predictors. Whereas, MFC significantly and strongly predicted task choice over and above NFC, *B* = 0.46, *SE B* = 0.05, β = 0.51, *t* = 9.15, *p* < 0.001, two-tailed, NFC was not significantly related to task choice, *B* = 0.14, *SE B* = 0.08, β = 0.11, *t* = 1.87, *p* = 0.06, two-tailed [overall model: *F*_(2,291)_ = 70.51, *p* < 0.001, $R_{\text{adj}}^{2}$ = 0.32]. In sum, Study 1 yielded initial evidence for the validity and utility of the MFC scale.

## Study 2

### Methods

#### Participants

The participants were recruited via non-public student mailing lists. In an email, recipients were asked to participate in an online study, and a link to the study was provided. Ninety-eight individuals clicked the link that led them to the welcome page of the study; 80 of them (60% female; *M*_age_ = 25.08 years; *SD*_age_ = 4.08) completed all measures (including questions about gender and age at the end of the study). Several individuals stopped their participation at some point during data collection. As the *N*s for the MFC scale and the validation measures therefore vary (see Table 2), so do the *N*s of the reported analyses (see the respective Table notes).

**Table 2:** Descriptive statistics and intercorrelations of the applied measures in study 2.

						**Intercorrelations**							
**Measure**	**Dimension of measurement**	***n***	***M***	***SD***	**α**	**1**	**2**	**3**	**4**	**5**	**6**	**7**	**8**
1. Motivation for cognition	State	87	3.72	1.29	0.92	–							
2. Need for cognition	Trait	82	4.82	0.77	0.86	0.51^***^	–						
3. Hope of success	Trait	89	3.26	0.44	0.73	0.23^*^	0.48^***^	–					
4. Fear of failure	Trait	89	2.39	0.64	0.81	−0.45^***^	−0.44^***^	−0.10	–				
5. Self-control capacity	State	88	4.50	1.16	0.88	0.59^***^	0.26^*^	0.04	−0.37^***^	-			
6. Subjective vitality	State	91	3.72	1.45	0.93	0.57^***^	0.28^*^	0.21	−0.32^**^	0.73^***^	-		
7. Positive affect	State	87	2.58	0.67	0.85	0.57^***^	0.29^**^	0.30^**^	−0.20	0.59^***^	0.70^***^	-	
8. Negative affect	State	87	1.56	0.66	0.88	−0.25^*^	−0.27^*^	−0.19	0.43^***^	−0.52^***^	−0.53^***^	−0.30^**^	-
9. Choice of task difficulty	State	82	3.43	1.41	–	0.43^***^	0.44^***^	0.37^***^	−0.25^*^	−0.42^***^	0.41^***^	0.24^*^	−0.28^*^

*Ns for intercorrelations: 82–89. Overall scores of a psychometric scale were obtained by averaging the responses to the scale items*.

* *p < 0.05, two-tailed*.

** *p < 0.01, two-tailed*.

*** *p < 0.001, two-tailed*.

#### Measures

In the following, we mention all measures that we applied. The measures were presented via computer, each appearing on a separate page. The computer software (Unipark) randomly determined the order of their appearance, except for the choice task and the demographic data questions which were presented at the end of the survey. The Cronbach's αs in the present study are shown in Table 2.

##### Motivation for Cognition and Need for Cognition

Motivation for cognition and NFC were measured with the same scales as in Study 1.

##### Hope of Success and Fear of Failure

We used the revised brief Achievement Motives Scale for German-speaking samples (Lang and Fries, 2006) to measure hope of success with five items (e.g., “I am attracted to situations allowing me to test my abilities”) and fear of failure with five items (e.g., “Even if nobody notices my failure, I'm afraid of tasks which I'm not able to solve”). The statements were answered on four-point Likert-type scales from 1 (*not true at all*) to 4 (*absolutely true*), and as to how they apply to one in general.

##### Self-Control Capacity

The German brief version of the State Self-Control Capacity Scale (Bertrams et al., 2011) was employed. The scale consists of 10 items (e.g., “I feel sharp and focused”) that the participants completed on Likert-type scales from 1 (*does not apply at all*) to 7 (*applies exactly*), with respect to the present moment.

##### Subjective Vitality

Participants indicated their momentarily perceived vitality on the six items of the German state version of the Subjective Vitality Scale (Bertrams et al., 2020). A sample item was “I feel alive and vital.” Answers were given on seven-point Likert-type scales from 1 (*does not apply at all*) to 7 (*applies exactly*).

##### Positive and Negative Affect

With the German adaptation of the Positive and Negative Affect Schedule (Krohne et al., 1996), we measured momentary mood. Participants indicated how they felt at the moment on 10 items for positive affect (e.g., “excited”) and another 10 items for negative affect (e.g., “nervous”). All 20 items were responded to on scales from 1 (*not at all*) to 5 (*extremely*).

##### Choice of Task Difficulty Item

After answering the motivational and affective measures, the participants were informed that the next page would contain five anagrams that they would be asked to solve. With the help of two examples, it was explained to them that an anagram is a scrambled word that has to be rearranged into a meaningful German word [e.g., EMRE to MEER (engl.: sea); EIGLESE to SEEIGEL (engl.: sea urchin)]. In addition, the participants read that it would be up to them to select their level of difficulty of the anagrams. There would be six levels of difficulty, depending on the number of letters each anagram word consists of, and higher levels would be mentally more effortful to solve. The participants then could choose between difficulty level A (three letters), B (four letters),…, and F (eight letters) (Note that unlike some countries, in Germany, grades are *not* assigned as letters between A and F but as numbers; therefore, for our German participants, the labeling of the difficulty levels was not confounded with the common evaluation of achievement). For the purpose of data analyses, we coded a selection of level A as 1, of level B as 2, and so forth. A translated version of this measure is presented in Appendix B.

After the participants had chosen one difficulty level and clicked on “continue”, they were debriefed on a newly appearing page that informed them that, in fact, no anagram task would take place because for the present study, it was only of interest as to how motivated people are at the moment to solve rather difficult anagrams. We informed them about a website that provides puzzles, including anagrams; after assessment of personal data and offered the respective weblink.

### Results and Discussion

Table 2 shows the descriptive statistics for the applied measures and how they were intercorrelated. Again, the MFC scale displayed good internal consistency (Cronbach's α = 0.92). As can be seen in Table 2, responses to the MFC scale were significantly related to the validity criteria, each in the expected direction. Thus, the bivariate correlations provide evidence for the validity of the MFC scale because all relationships were predefined from a theoretical base.

The MFC scale was superior to the other applied trait and state measures in predicting anagram task choice, as multiple regression analyses revealed (see Tables 3, 4). As expected, over and above trait measures, MFC significantly predicted the cognitive effort in an ostensibly subsequent task that participants were motivated to invest in (see Table 3). In contrast, the significant bivariate relations NFC and achievement motives had with task choice (Table 2) vanished when MFC was accounted for in the same model; overall model: *F*_(4,77)_ = 7.53, *p* < 0.001, $R_{\text{adj}}^{2}$ = 0.24, two-tailed. The trait measures including NFC may be seen as distal measures of cognitive motivation and their bivariate relations to task choice may be attributable to the variance they share with MFC.

**Table 3:** Multiple regression analysis for predicting choice of task difficulty by motivation for cognition and trait measures in study 2.

**Predictor**	***B***	***SE B***	**β**	***t***	***p* ^a^**
Motivation for cognition	0.29	0.13	0.26	2.23	0.03
Need for cognition	0.35	0.24	0.19	1.47	0.15
Hope of success	0.66	0.34	0.21	1.92	0.06
Fear of failure	−0.05	0.25	−0.02	−0.21	0.83

*N = 82*.

a *Two-tailed*.

**Table 4:** Multiple regression analysis for predicting choice of task difficulty by motivation for cognition and other state measures in study 2.

**Predictor**	***B***	***SE B***	**β**	***t***	***p* ^a^**
Motivation for cognition	0.32	0.15	0.29	2.15	0.04
Self-control capacity	0.21	0.19	0.17	1.10	0.28
Subjective vitality	0.21	0.17	0.22	1.25	0.21
Positive affect	−0.41	0.31	−0.20	−1.36	0.18
Negative affect	−0.11	0.26	−0.05	−0.42	0.68

*N = 82*.

a *Two-tailed*.

An additional multiple regression analysis showed that the MFC scale also predicted the chosen cognitive effort in the anagram task over and above the other state measures (see Table 4). The relations of self-control capacity, subjective vitality, and mood with task choice (Table 2) did not hold over and above MFC. The overall model was significant; *F*_(5,76)_ = 5.21, *p* < 0.001, $R_{\text{adj}}^{2}$ = 0.21, two-tailed. Thus, in support of its validity, the MFC scale was the state measure that best predicted a behavioral indicator of cognitive motivation, which was independent from momentary experience not directly defining cognitive motivation (e.g., subjective vitality).

## General Discussion

The aim of the present work was to find evidence that a state measure of the momentary motivation to engage in effortful cognition would usefully add to the existing measure of trait NFC. For this purpose, we developed the MFC scale and intended to show initial evidence that state cognitive motivation can reliably and validly be measured. The central findings from two studies can be summarized as follows. The MFC scale captures a unidimensional construct and is a reliable measure in terms of internal consistency. Furthermore, the scale is valid in terms of being theoretically meaningfully related to other measures—that is, hope of success and fear of failure (Lang and Fries, 2006), self-control capacity (Bertrams et al., 2011), subjective vitality (Bertrams et al., 2020), positive and negative affect (Krohne et al., 1996), NFC (Bless et al., 1994), and choice of task difficulty. Finally, the MFC scale is superior to related trait and state measures in predicting momentary motivation to engage in cognitive effort. In sum, our findings preliminarily indicate that the new MFC scale is a psychometrically sound measure.

There are numerous ways to apply the MFC scale in research and practice. For instance, the effect of low momentary relative to dispositional motivation to engage in cognitive effort on using stereotypes (Dudley and Harris, 2003) may be more pronounced. For several reasons, someone who is usually highly motivated for cognitive effort (i.e., high in NFC) might not be so during each experimental session; the reverse may also be true for someone low in NFC. Therefore, application of the MFC scale for measuring state cognitive motivation may enable researchers to test their respective hypotheses with more statistical power. Further, since intrinsically motivated learning is highly affected by a large set of situational and intrapersonal variables (Heckhausen and Heckhausen, 2018), motivational shifts toward and against cognitive effort may occur along with a variety of combined factors given the specific learning situation. Eventually, a state measure assessing student's momentary motivation for cognitive effort may be useful in school intervention studies. In educational counseling or clinical therapy, the MFC scale may function as a control of testees' motivation to complete cognitively effortful diagnostic instruments (e.g., tests of cognitive abilities).

This leads us to an interesting point. The relationship between NFC and intelligence has usually been found to be small, if existent at all (Cacioppo et al., 1996; Fleischhauer et al., 2010; Preckel, 2014). This has been interpreted such that NFC is a motivational—rather than an ability—construct (Cacioppo et al., 1996). It is, however, quite implausible that the motivation to spend cognitive effort does not affect how one performs in a cognitively challenging test. The lack of evidence for a substantial relation between cognitive motivation and intelligence may, in part, be attributable to the unequal dimensions on which both variables were assessed. Cognitive motivation was measured on the trait level (i.e., with the NFC scale), whereas intelligence was inferred from a specific performance situation. Possibly, the relationship between cognitive motivation and cognitive ability is larger than previously found when the dimensions of assessment match (i.e., when the MFC scale is applied directly before the intelligence test). In this context, one might also want to address the question of how typical vs. maximal performance in intelligence tests is related to state motivation for cognitive effort similar to previous studies of NFC and Motivation (e.g., Klehe and Anderson, 2007; Von Stumm, 2013). Examining the relation between the momentary motivation to invest cognitive effort and intelligent performance is an exciting direction for future research.

Furthermore, one can assume that such a measure could serve well in diary or experience sampling designs. In a diary study, for example, the use of the MFC scale may provide insights into how motivation for cognitive effort manifests itself over different times of the day, in various working group constellations, or in relation to different types of tasks and their specific characteristics. In sum, future research could aim to investigate structural and situational factors in the work or study environment linked to MFC and its relevant outcomes.

Our findings also add to the evidence of good psychometric properties of the NFC scale (e.g., Bless et al., 1994; Cacioppo et al., 1996; Bertrams and Dickhäuser, 2010). Similar to the MFC scale, the NFC scale was meaningfully related to all validity criteria in the present studies. To examine relationships on the dispositional level, the NFC scale—rather than the MFC scale—may be the appropriate measure. Moreover, when there is a larger time lag between the assessment of cognitive motivation and of other variables in a study, the NFC scale may be of exceptional use. For instance, researchers may sometimes like to measure cognitive motivation several weeks prior to an experiment to avoid having participants suspect the underlying hypothesis. In such cases, it would not make much sense to apply a state measure of motivation for cognitive effort, but rather the NFC scale. However, when it is important to assess cognitive motivation as it exists in a concrete situation, the MFC scale may often be the more accurate measure.

Some limitations of the present study deserve attention. For instance, a criterion we didn't address was the MFC scale's sensitivity to change (e.g., its responsiveness; Husted et al., 2000). One can assume, that a person's MFC changes on a daily basis due to mental fatigue fluctuations (for a detailed overview on fatigue see, van der Linden, 2011) or simply not being in the mood for applying cognitive effort. Therefore, further research is needed to establish the MFC scale's sensitivity regarding changes in individuals' willingness to exert cognitive effort in different contexts. This could either be realized by repeated measures over time (e.g., Wilhelm and Schoebi, 2007) or by experimentally manipulating conditions, e.g., mood induction procedures (MIP) (see Westermann et al., 1996). In addition, longitudinal designs would allow to capture the trait- and state-specific components of MFC, e.g., by applying latent state–trait (LST) models (Steyer et al., 1999). Another important direction for future research could be to highlight the state–trait difference by showing significant temporal variance in MFC and relative stability in NFC. Last, generalizability of findings is limited to the characteristics of the sample studied, namely German university students of a certain age group with comparable educational backgrounds. It remains to be investigated whether the MFC scale is also a valid and reliable instrument in non-student samples or among children and adolescents as has been done previously with the NFC-Scale (e.g., Preckel, 2014; Keller et al., 2019).

## Conclusions

Even though we consider our findings preliminary, this article provides initial evidence for a valid and reliable measurement of MFC from a state perspective as well as useful recommendations for scientific implementation.

## Data Availability Statement

The raw data supporting the conclusions of this article will be made available by the authors, without undue reservation.

## Ethics Statement

Ethical review and approval was not required for the study on human participants in accordance with the local legislation and institutional requirements. The participants provided their written informed consent to participate in this study.

## Author Contributions

TM and AB: developed the study concept and design and collected data. AB and MB: analyzed, interpreted the data, and prepared the draft manuscript. AK and TM: provided critical revisions. All authors contributed meaningfully to the paper, approve the final version to be published, and agree to be accountable for all aspects of the work in ensuring that questions related to the accuracy or integrity of any part of the work are appropriately investigated and resolved.

## Conflict of Interest

The authors declare that the research was conducted in the absence of any commercial or financial relationships that could be construed as a potential conflict of interest.

## Publisher's Note

All claims expressed in this article are solely those of the authors and do not necessarily represent those of their affiliated organizations, or those of the publisher, the editors and the reviewers. Any product that may be evaluated in this article, or claim that may be made by its manufacturer, is not guaranteed or endorsed by the publisher.
