# Supplementary material for: Measuring Motivation for Cognitive Effort as State
Source: Front Psychol. 2021 Dec 9;12:785094. doi: 10.3389/fpsyg.2021.785094 (PMC8695725; doi:10.3389/fpsyg.2021.785094)
Supplement: Supplementary file 1 [file Data_Sheet_1.docx]

**Appendix A**

Choice of task difficulty item in Study 1:

If you could choose the difficulty level of a short task directly following this questionnaire yourself, which difficulty level would you choose? (Please tick one level of difficulty).

❒ Difficulty level 1: very low mental effort required

❒ Difficulty level 2: low mental effort required

❒ Difficulty level 3: rather low mental effort required

❒ Difficulty level 4: rather high mental effort required

❒ Difficulty level 5: high mental effort required

❒ Difficulty level 6: very high mental effort required

**Appendix B**

Choice of task difficulty item in Study 2:

Please solve five anagrams on the following page. An anagram is a mental problem-solving task. It is about forming a meaningful German word from jumbled letters. All letters from the letter string must be used. For example, the word MEER [engl.: sea] can be formed from the letter string EMRE by rearranging the letters; the word SEEIGEL [engl.: sea urchin] can be formed from the letter string EIGLESE.

You determine the level of difficulty yourself. There are six levels of difficulty. The higher the difficulty level, the more letters the anagrams contain and the more mentally demanding it is to solve the anagrams.

Please choose your difficulty level and click on "Next".

❒ 3 letters per anagram (level A)

❒ 4 letters per anagram (level B)

❒ 5 letters per anagram (level C)

❒ 6 letters per anagram (level D)

❒ 7 letters per anagram (level E)

❒ 8 letters per anagram (level F)
